# Landscape of germline *BRCA1/BRCA2* variants in breast and ovarian cancer in Peru

**DOI:** 10.3389/fonc.2023.1227864

**Published:** 2023-08-17

**Authors:** Yomali Ferreyra, Gina Rosas, Alicia M. Cock-Rada, Jhajaira Araujo, Leny Bravo, Franco Doimi, Jhoysi Casas, María de los Ángeles Clavo, Joseph A. Pinto, Carolina Belmar-López

**Affiliations:** ^1^ Departamento de Bioingeniería, Universidad de Ingenieria y Tecnología, Lima, Peru; ^2^ Departamento de Patología, Insituto Nacional de Enfermedades Neoplásicas, Lima, Peru; ^3^ Departmento de Oncología Médica, Instituto de Cancerología Las Américas - Auna, Medellín, Colombia; ^4^ Centro de Investigación Básicas y traslacional, Auna Ideas, Lima, Peru; ^5^ Escuela Profesional de Medicina Humana-Filial Ica, Universidad Privada San Juan Bautista, Ica, Peru; ^6^ Oncogenomics, Auna, Lima, Peru; ^7^ Facultad de Medicina Humana, Universidad Nacional San Luis Gonzaga, Ica, Peru

**Keywords:** breast cancer, ovarian cancer, BRCA1, BRCA2, germline mutation

## Abstract

**Background:**

There is an increasing amount of data from Latin America on the characterization of BRCA variants; however, there is limited information from Peru. We conducted a retrospective study to describe germline pathogenic/likely pathogenic(P/LP) variants and variants of uncertain/unknown significance (VUS) in the BRCA1 and BRCA2 genes in Peru, in patients with breast and ovarian cancer, candidates for treatment with poly (adenosine diphosphate–ribose) polymerase (PARP) inhibitors.

**Methods:**

The patients were evaluated during the period 2019-2021. Genomic DNA was isolated from peripheral blood samples and targeted sequencing was performed using the Ampliseq BRCA panel. Genetic variant interpretation was carried out in accordance with the recommendations of the American College of Medical Genetics and ClinVar. During this period, 525 patients (143 with breast cancer and 382 with ovarian cancer) were studied.

**Results:**

We found that 14.7% (21/143) of breast cancer patients and 20.7% (79/382) of ovarian cancer patients were carriers of P/LP variants in BRCA1/2. The most frequent pathogenic variants detected in BRCA1 were c.2105dupT (BIC: 2224insT, n=12, 18.75%), c.68_69delAG (BIC: 185delAG, n=6, 9.38%), c.140G>T and c.815_824dupAGCCATGTGG (n=5, 7.81%), while in BRCA2 were c.8023A>G (n=6, 16.67%), c.6024dupG (BIC: 6252insG, n=4, 11.11%), and c.9235delG (BIC: 9463delG, n=3, 8.33%). Regarding VUS, we found that 6.99% (10/143) of breast cancer patients and 7.33% (28/382) of ovarian cancer patients were carriers of a VUS in BRCA1/2. For BRCA1, the most frequent VUS was c.93C>G (n=2), and for BRCA2, c.5465A>T (n=4), c.3101T>C (n=3), c.205C>A and c.437T>C (n=2).

**Conclusion:**

We found a frequency of 14.7% germline mutations in breast cancer patients and 20.7% in ovarian cancer patients. The most recurrent mutations were BRCA1 c.2105dupT and BRCA2 c.8023A>G. We found that BRCA2 c.8023A>G, c.6024dupG, and c.9235delG were not previously reported in Peruvian patients. BRCA1 c.2344dupA is a novel mutation that has not been previously reported in any database. The frequency of VUS in our cohort was 7.2%.

## Background

Breast and ovarian cancer are highly prevalent malignancies in Latin America, where more than 50 thousand patients die yearly from these pathologies. It is estimated that 5-10% of breast cancer (BC) and 10-15% of ovarian cancer (OC) are attributed to hereditary breast and ovarian cancer (HBOC). Mutations in the *BRCA1* and *BRCA2* genes are the best-known genetic risk factors for breast and ovarian cancer (1–3).

Detecting *BRCA1/2* mutations allows crucial and challenging decisions regarding cancer prevention or risk reduction, early detection and pharmacological management. Germline *BRCA1/2* variants cause hereditary breast and ovarian cancer syndrome and confer a lifetime risk of 38-87% of developing breast cancer in women and 16.5-63% of developing ovarian cancer (4). Germline *BRCA1/2* mutations are present in approximately 5% of all breast patients and 15% of all ovarian cancer patients (5, 6). Breast tumors with *BRCA1/2* mutation carriers are more frequently HER2 negative, and triple-negative tumors (TNBC) account for approximately 68% of *BRCA1* tumors (7). High-grade serous ovarian cancers are more commonly associated with *BRCA1/2* mutations (8).

*BRCA1/2* are tumor suppressor genes that participate in the homologous recombination repair of double-strand DNA damage. *BRCA1/2* defective cells are more sensitive to Poly(ADP-ribose) polymerase (PARP), an enzyme involved in base excision repair, a vital component of single-strand break repair (SSB). PARP inhibitors can increase SSB, which are converted to double-strand DNA breaks (DSBs) during replication. In defective *BRCA1/2* cells, these DSBs are not effectively repaired, leading to chromosomal instability, cell cycle arrest, and subsequent apoptosis, a mechanism known as synthetic lethality (9)

Several PARP inhibitors have been developed to treat patients with breast, ovarian, pancreatic, and prostate cancer with *BRCA1/2* mutations. OlympiAD, a phase III randomized, controlled, open-label study trial, compared Olaparib, a PARP inhibitor, with non-platinum chemotherapy (CT) in metastatic breast cancer patients with germline *BRCA*1/2 mutations. Progression-free survival (PFS) was higher in patients receiving Olaparib than in those receiving CT (7.0 vs 2.2 months). The hazard ratio (HR) for disease progression or death was 0.58 (95% CI 0.43-0.80: P< 0.001). The response rate was 59.9% in the Olaparib group and 28.8% in the CT group. An improvement in PFS was observed in all subtypes and was higher in TNBC patients (10).

The randomized phase III trial, EMBRACA, compared another PARP inhibitor, Talazoparib, with single-agent CT in patients with advanced breast cancer with *BRCA1/2* mutations. The median PFS was longer in the Talazoparib group than in the control group (8.6 months [95% CI, 7.2-9.3] vs 5.6 months [95% CI, 4.2-6.7]; HR=0.54 [95% CI 0.41-0.71; P < 0.001] for disease progression or death. Based on these two trials, the FDA approved Olaparib and Talozaparib for HER2-negative patients with *BRCA1/2* mutations, and the NCCN guidelines recommend testing for *BRCA1/2* mutations in all patients with TNBC or metastatic breast cancer to identify candidates for PARP inhibitor therapy (11).

For ovarian cancer, germline testing for *BRCA*1/2 is informative for selecting patients for PARP inhibitor therapy. Around 50% of ovarian tumors are homologous recombination deficient (HRD) and 20% are associated with *BRCA1/2* mutations, germline (14%) or somatic (6%) (6). PARP inhibitor treatment is recommended for maintenance therapy for stage II-IV disease or persistent or recurrent disease in BRCA*1/2* mutated ovarian cancer (12). The SOLO-1 phase III trial showed an improvement in 3-year PFS with Olaparib versus Placebo as maintenance therapy for patients with a germline *variant BRCA1/2*, who had a complete or partial response (CR/PR) after platinum-based first-line chemotherapy (60% vs. 27%; P<0.0001); HR=0.30 [95% CI, 0.23-0.41] (13). Other phase III studies with PARP inhibitor, PAOLA-1: Olaparib + Bevacizumab versus Placebo + Bevacizumab for maintenance therapy for patients with advanced disease who had CR/PR after platinum-based first-line chemotherapy, or PRIMA: Niraparib versus Placebo as maintenance therapy for patients with CR/PR after platinum first-line chemotherapy showed a notable improvement in median PFS, especially in patients with a germline *BRCA*1/2 mutation or HRD (22.1 vs 16.6 months and 21.9 months vs 10.4 months, respectively) (14, 15).

There is increasing information on the molecular epidemiology of *BRCA1/2* genes in Latin America and several founder mutations have been identified (16). Founder mutations in Latin America have been reported in Mexico (*BRCA1* del exons 9-12), Brazil (*BRCA1* 5382insC and *BRCA2* c.156_157insAlu), and Colombia (*BRCA1* 3450del4, A1708E and *BRCA2* 3034del4) (17).

Unfortunately, there is a gap in information about the genetic epidemiology of breast and ovarian cancers in Peru. A limited number of publications describe the frequency of *BRCA1* and *BRCA2* mutations in Peru with studies with small sample sizes and in a particular group of patients (18). Here, we describe the frequency and spectrum of genetic variants (pathogenic/likely pathogenic P/LP and VUS) in *the BRCA1/2* genes in patients with BC and/or OC, profiled during 2019-2021 in a reference laboratory in Peru, for the identification of PARP inhibitor candidates.

## Methods

### Study population

The study included all BC and OC patients, referred to Oncogenomics Laboratory (Lima, Peru) from 2019 to 2021, to identify candidates for PARP inhibitors treatment. The histological tumor types, hormone receptor and human epidermal growth factor receptor 2 (HER2) statuses were recorded.

### *BRCA1/2* mutation screening

Peripheral blood samples were taken to assess mutations in *the BRCA1/2* genes. DNA was extracted using the ReliaPrep Blood gDNA Miniprep System or the ReliaPrep FFPE gDNA Miniprep System (Promega, Madison, USA) according to the manufacturer’s protocol. DNA concentration was determined by fluorometric quantitation using a Qubit 4.0 fluorimeter with Qubit dsDNA HS ASSAY KIT (Invitrogen, USA). The panel targets single nucleotide variants (SNVs) and insertion/deletions (indels) in all exonic regions of the *BRCA1* and *BRCA2* genes and flanking intronic sequences. A commercial reference standard, Horizon Quantitative Multiplex Reference Standard HD810 was tested to validate the performance of NGS that detect mutations. Libraries were prepared using the Ampliseq *BRCA* Panel and Ampliseq Library PLUS (Illumina, San Diego, USA) following the manufacturer’s protocol without modifications using 10 ng of input DNA per sample. The multiplex polymerase chain reaction (PCR) was performed in 18 cycles. Sequencing adapters with unique indexes (AmpliSeq CD Indexes Set A for Illumina) were ligated to the amplification products and purified using Agencourt AmpureXP beads (Beckman Coulter, CA, USA) according to the manufacturer’s instructions. Libraries with 2nM molarities were subjected to clustering using a standard flow cell and sequenced on the Illumina MiSeq platform using the MiSeq Reagent Micro Kit v2 (300 cycles). Raw data were processed automatically in the BaseSpace Sequence Hub (Illumina) and aligned with the hg19 reference genome. An average of 95.7% (91.7– 99.8%) on-target reads, 96.6% (94.7 – 98.6%) read uniformity and 50X average coverage per sample were obtained.

### Mutation nomenclature and classification

BaseSpace Variant Interpreter (Illumina) annotated and interpreted genetic variants. Genetic variants were annotated under the nomenclature of the Human Genome Variation Society (HGVS). Interpretation was made using the single nucleotide polymorphism database (dbSNP), breast cancer information core (BIC) and Clinvar database. The Integrative Genomics Viewer was applied to visualize the variants. All identified variants were checked with Varsome (Saphetor, Swiss).

Interpreting the pathogenicity of the variants followed the latest recommendations of the American College of Medical Genetics (ACMG) and ClinVar. In cases of conflicting interpretations of pathogenicty (classified as pathogenic by at least one database and benign, likely benign, or VUS by others) between ACMG and ClinVar, ACMG classification was used. All new variants (missense, in frame and splicing) not yet reported in the databases consulted, were also considered VUS. Benign variants were not reported.

### Statistical analysis

We conducted a descriptive analysis. The frequency of variants identified in the *BRCA1/2* genes was calculated using RStudio. Packages such as *sf, ggplot2* and *ggrepel* were installed to obtain geographical maps of Peru, to observe the distribution of pathogenic variants according to the place of birth of the patients.

## Results

### General characteristics of patients

**Figure 1:**
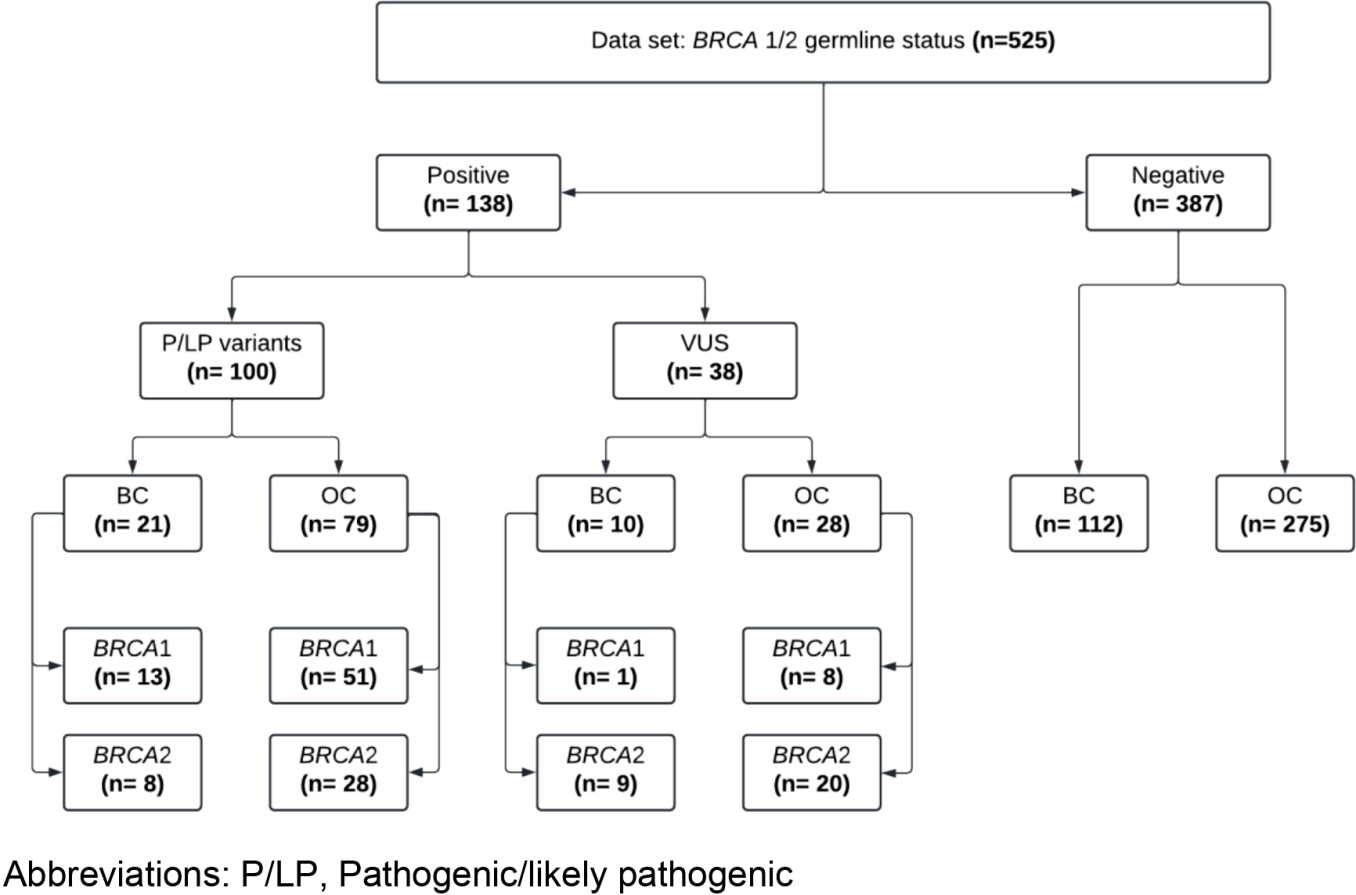
Flowchart showing results and characteristics of patients with breast and ovarian cancer included in this study. P/LP, Pathogenic/likely pathogenic.

A total of 525 patients were included in this study (143 with BC and 382 with OC). Most of the patients were born in metropolitan Lima (58.1%), followed by patients from the coast (excluding metropolitan Lima, 28.0%), highlands (11.6%) and jungle (2.1%). Regarding the clinical characteristics of BC patients, 21.7% (n=31) had HER2-negative and hormone receptor positive tumors, while 78.3% (n=112) of the patients had HER2-negative and hormone receptor negative tumors (TNBC). Most of the OC (90.8%) were high-grade serous carcinomas. The frequency of clinical stage III was 33.6% and 50%, and clinical stage IV was 66.4% and 50% for BC and OC, respectively (**Table 1**). The prevalence of patients with P/LP germline variants in *BRCA1/2* was 19.0% (n=100/525). No patient had more than one P/LP variant. The percentage of BC patients who carried germline *BRCA1/2* P/LP variants was 14.7% (n=21/143) with 61.9% (n=13/21) of them carrying variants in *BRCA1* and 38.1% (n=8/21) in *BRCA2* (**Figure 1**). In total, 81% (n=17/21) of patients with *BRCA1/2* P/LP variants were TNBC and 19.1% (n=4/21) were positive for hormone receptors. Furthermore, 38.1% (n=8/21) were reported in clinical stage III and 61.9% (n=13/21) in clinical stage IV. Although we had no information about their age at the time of diagnosis, the mean age at molecular diagnosis was 48.63 years (range: 30-74 years).On the other hand, 20.7%(n=79/382) of OC patients had germline *BRCA1/2* P/LP variants with 64.8% (n=51/79) of them in *BRCA1* and 35.44% (n=28/79) in *BRCA2*. Regarding the histological subtypes of *BRCA1/2* OC patients, it was observed that 91.1% (n=72/79) were characterized by high-grade serous carcinoma, followed by endometrioid carcinoma (n=5/79, 6.3%) and clear cell carcinoma (n=2/79, 2.53%). The clinical stages were distributed in 49.4% (n=39/79) stage III and 50.6% (n=40/79) stage IV. Furthermore, only 77 patients had their age at molecular diagnosis available, so the mean age was 54.36 years (range: 34-77).

**Table 1:** Clinicopathological characteristics of the general population.

Clinical characteristics		BC % (n=143)	OC % (n=382)	All patients (n=525)
**BRCA1/2 status**	**POSITIVE**	21.7 (31)	28.0 (107)	26.3 (138)
	P/LP	14.7 (21)	20.7 (79)	19.0 (100)
	VUS	7.0 (10)	7.3 (28)	7.2 (38)
	**NEGATIVE**	78.3 (112)	72.0 (275)	73.7 (387)
**Clinical stage**	III	33.6 (48)	50.0 (191)	45.5 (239)
	IV	66.4 (95)	50.0 (191)	54.5 (286)
**IHC subtype**	HER2- HR-	78.3 (112)	—	21.3 (112)
	HER2- HR+	21.7 (31)	—	5.9 (31)
**Histologic subtype**	High grade serous	—	90.8 (347)	66.1 (347)
	Low grade serous	—	0.8 (3)	0.6 (3)
	Clear cell carcinoma	—	3.7 (14)	2.6 (14)
	Endometrioid carcinoma	—	3.9 (15)	2.8 (15)
	Others	—	0.8 (3)	0.6 (3)
**Birthplace**	Metropolitan Lima	64.3 (92)	55.8 (213)	58.1 (305)
	Coast^*^	25.2 (36)	29.1 (111)	28.0 (147)
	Highlands	8.4 (12)	12.8 (49)	11.6 (61)
	Jungle	1.4 (2)	2.4 (9)	2.1 (11)
	Unknown	0.7 (1)	0.0 (0)	0.2 (1)

*Excluding Metropolitan Lima.

### Germline *BRCA1* pathogenic/likely pathogenic variants

The study identified 33 different P/LP germline variants in *BRCA1* gene in 64 patients, finding 20.3% (n=13/64) in BC and 79.9% (n=51/64) in OC. The most frequent variants in *BRCA1* were c.2105dupT (BIC: 2224insT, n=12, 18.75%), c.68_69delAG (BIC: 185delAG, n=6, 9.38%), c.140G>T and c.815 _824dupAGCCATGTGG (n=5, 7.81%) (**Table 2**). Most variants were previously reported, except *BRCA1* c.2344dupA (n=2, 2.74%), which was not reported in any database. These patients were born in Lima and Arequipa.

**Table 2:** Pathogenic/likely pathogenic germline *BRCA1* variants found in our study.

HGVSc Nomenclature	HGVSp Nomenclature	Effect	Exon	Frequency % (n=64)	Tumor type	Mutation type	Reference
2105dupT	Leu702Phefs*10	L702Ffs*10	10	18.75% (12)	OC (9), TNBC (3)	inframe insertion	ACMG
68_69delAG	Glu23Valfs*17	E23Vfs*17	2	9.38% (6)	OC (5), TNBC (1)	inframe deletion	ACMG/ClinVar
140G>T	Cys47Phe	C47F	4	7.81% (5)	OC (4), TNBC (1)	missense	ACMG/ClinVar
815_824dupAGCCATGTGG	Thr276Alafs*14	T276Afs*14	10	7.81% (5)	OC	inframe insertion	ACMG/ClinVar
5123C>A	Ala1708Glu	A1708E	17	6.25% (4)	OC (3), TNBC (1)	missense	ACMG/ClinVar
1961delA	Lys654Serfs*47	K654Sfs*47	10	4.69% (3)	OC (2), BC (HER2- RH+, 1)	inframe deletion	ACMG/ClinVar
2344dupA	Ser782Lysfs*8	S782Kfs*8	10	3.13% (2)	OC	inframe insertion	ACMG/ClinVar
5251C>T	Arg1751*	R1751*	19	3.13% (2)	OC (1), TNBC (1)	missense	ACMG/ClinVar
62_66delTCTTA	Ile21Argfs*18	I21Rfs*18	2	1.56% (1)	OC	inframe deletion	ACMG
211A>G	Arg71Gly	R71G	4	1.56% (1)	OC	missense	ACMG/ClinVar
250G>T	Glu84*	E84*	5	1.56% (1)	OC	missense	ACMG/ClinVar
798_799delTT	Ser267Lysfs*19	S267Kfs*19	10	1.56% (1)	OC	inframe deletion	ACMG/ClinVar
928C>T	Gln310*	Q310*	10	1.56% (1)	TNBC	missense	ACMG/ClinVar
1088delA	Asn363Ilefs*11	N363Ifs*11	10	1.56% (1)	OC	inframe deletion	ACMG/ClinVar
1700dupA	Asn567Lysfs*3	N567Kfs*3	10	1.56% (1)	OC	inframe insertion	ACMG/ClinVar
2215A>T	Lys739*	K739*	10	1.56% (1)	OC	missense	ACMG/ClinVar
2493T>G	Tyr831*	Y831*	10	1.56% (1)	OC	stop gained	ACMG
2544delA	Glu849Lysfs*44	E849Kfs*44	10	1.56% (1)	OC	inframe deletion	ACMG
2649_2650delAA	Thr884Ilefs*18	T884Ifs*18	10	1.56% (1)	OC	inframe deletion	ACMG/ClinVar
2766delA	Val923Leufs*77	V923Lfs*77	10	1.56% (1)	OC	inframe deletion	ACMG/ClinVar
2839A>T	Lys947*	K947*	10	1.56% (1)	OC	missense	ACMG/ClinVar
2901_2902dupTC	Pro968Leufs*33	P968Lfs*33	10	1.56% (1)	OC	inframe insertion	ACMG/ClinVar
2913delT	His971Glnfs*29	H971Qfs*29	10	1.56% (1)	OC	inframe deletion	ACMG
3005delA	Asn1002Thrfs*22	N1002Tfs*22	10	1.56% (1)	OC	inframe deletion	ACMG/ClinVar
3455delA	Asp1152Alafs*3	D1152Afs*3	10	1.56% (1)	TNBC	inframe deletion	ACMG/ClinVar
3626T>A	Leu1209*	L1209*	10	1.56% (1)	OC	missense	ACMG
3800T>G	Leu1267*	L1267*	10	1.56% (1)	OC	missense	ACMG/ClinVar
del exon 11			11	1.56% (1)	OC	deletion variant	
4327C>T	Arg1443*	R1443*	12	1.56% (1)	TNBC	missense	ACMG/ClinVar
4484G>T	Arg1495Met	R1495M	13	1.56% (1)	OC	missense	ACMG/ClinVar
5158C>T	Arg1720Trp	R1720W	18	1.56% (1)	OC	missense	ACMG/ClinVar
5266dupC	Gln1756Profs*74	Q1756Pfs*74	19	1.56% (1)	TNBC	inframe insertion	ACMG/ClinVar
67_75delGAGTGTCCC	Glu23_Pro25del	E23_P25del	2	1.56% (1)	TNBC	inframe deletion	ACMG/ClinVar*

**Figure 2:**
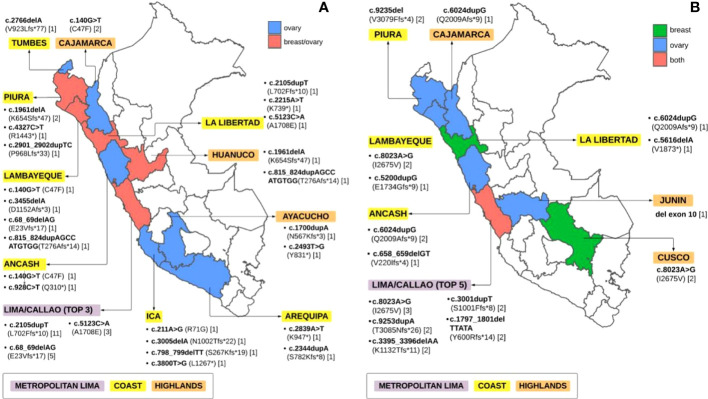
Pathogenic/likely pathogenic germline *BRCA1/2* variants according to the place of birth **(A)** Germline *BRCA1* LP/P variants **(B)** Germline *BRCA2* LP/P variants.

**Figure 3:**
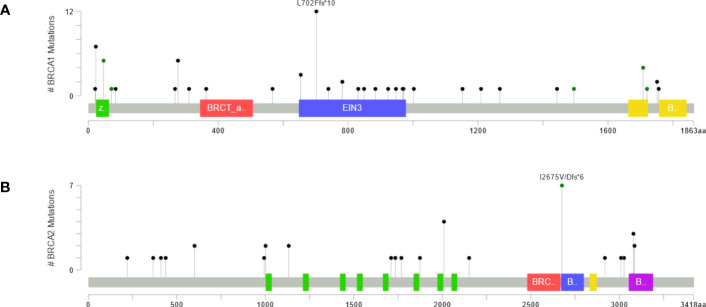
Maps presenting counts of pathogenic and likely pathogenic variants sites in **(A)** Brca1 and **(B)** Brca2 proteins.

Furthermore, *BRCA1* P/LP variants were observed mainly in exon 10 (n=20/33), exon 19 (n = 3/33), and exon 2 and 4 (n=2/33). Most of the P/LP variants were missense (n=13/33) and in frame deletion variants (n=12/33), followed by frame insertions (n=6/33) and stop gained variants (n=1/33) (**Table 2**). There was only one case with a large deletion, specifically a deletion of exon 11 (n=1/33). Patients with P/LP variants in the *BRCA1* gene were born primarily in Metropolitan Lima (n=37/64), the coast (n=21/64) and the highlands (n=6/64). The most frequent P/LP variants in *BRCA1*, c.2105dupT (n=11) and c.68_69delAG (n=5) were found predominantly in patients born in Metropolitan Lima (**Figure 2A**). The position of P/LP variants in relation to the Brca1 protein is shown in **Figure 3A**.

### Germline *BRCA2* pathogenic/likely pathogenic variants

We found 22 different P/LP variants in *BRCA2* among 36 patients, 22.2% (n=8/36) in BC and 77.8% (n=28/36) in OC. The most frequent mutations in *BRCA2* were c.8023A>G (n=6, 16.67%), c.6024dupG (BIC: 6252insG, n=4, 11.11%), and c.9235delG (BIC: 9463delG, n=3, 8.33%) (**Table 3**). Likewise, most of the *BRCA2* P/LP variants were found only in OC patients (n=14/22, 63.63%), followed by both OC and BC patients (n=4/22, 18.18%) and only BC patients (n=4/22, 18.18%). Furthermore, *BRCA2* variants were mainly reported in exon 11 (n=9/22, 40.91%), predominantly inframe deletions (n=10/22), followed by inframe insertions (n=6/22) and missense variants (n=5/22) (**Table 3**). Only one large deletion was identified in exon 10. Patients with P/LP variations in *BRCA2* were born in Metropolitan Lima (n = 24/36), on the coast (n=9/36) and in the highlands (n=3/36) (**Figure 2B**). The position of P/LP variants in relation to the Brca*2* protein is shown in **Figure 3B**.

**Table 3:** Pathogenic/likely pathogenic germline *BRCA2* variants found in our study.

HGVSc Nomenclature	HGVSp Nomenclature	Effect	Exon	Frequency % (n=36)	Tumor type	Mutation type	Reference
8023A>G	Ile2675Val	I2675V	18	16.67% (6)	OC (5), TNBC (1)	missense	ACMG/ClinVar
6024dupG	Gln2009Alafs*9	Q2009Afs*9	11	11.11% (4)	OC (3), BC (HER2- RH+, 1)	inframe insertion	ACMG
9235delG	Val3079Phefs*4	V3079Ffs*4	24	8.33% (3)	OC	inframe deletion	ACMG/ClinVar
1797_1801delTTATA	Tyr600Argfs*14	Y600Rfs*14	10	5.56% (2)	OC (1), TNBC (1)	inframe deletion	ACMG/ClinVar
3001dupT	Ser1001Phefs*8	S1001Ffs*8	11	5.56% (2)	OC	inframe insertion	ACMG/ClinVar
3395_3396delAA	Lys1132Thrfs*11	K1132Tfs*11	11	5.56% (2)	OC (1), TNBC (1)	inframe deletion	ACMG
9253dupA	Thr3085Asnfs*26	T3085Nfs*26	24	5.56% (2)	OC	inframe insertion	ACMG/ClinVar
658_659delGT	Val220Ilefs*4	V220Ifs*4	8	2.78% (1)	OC	inframe deletion	ACMG/ClinVar
1097T>G	Leu366*	L366*	10	2.78% (1)	OC	missense	ACMG/ClinVar
1228A>T	Lys410*	K410*	10	2.78% (1)	OC	missense	ACMG
1310_1313delAAGA	Lys437Ilefs*22	K437Ifs*22	10	2.78% (1)	OC	inframe deletion	ACMG/ClinVar
del exon 10			10	2.78% (1)	OC	deletion variant	
2979G>A	Trp993*	W993*	11	2.78% (1)	TNBC	missense	ACMG/ClinVar
5130_5133delTGTA	Tyr1710*	Y1710*	11	2.78% (1)	OC	inframe deletion	ACMG/ClinVar
5200dupG	Glu1734Glyfs*9	E1734Gfs*9	11	2.78% (1)	OC	inframe insertion	ACMG
5303_5304delTT	Leu1768Argfs*5	L1768Rfs*5	11	2.78% (1)	OC	inframe deletion	ACMG/ClinVar
5616delA	Val1873*	V1873*	11	2.78% (1)	TNBC	inframe deletion	ACMG/ClinVar
6450dupA	Val2151Serfs*25	V2151Sfs*25	11	2.78% (1)	OC	inframe insertion	ACMG
8021dupA	Ile2675Aspfs*6	I2675Dfs*6	18	2.78% (1)	BC (HER2- RH+)	inframe insertion	ACMG/ClinVar
8752G>T	Glu2918*	E2918*	21	2.78% (1)	BC (HER2- RH+)	missense	ACMG
9026_9030delATCAT	Tyr3009Serfs*7	Y3009Sfs*7	23	2.78% (1)	OC	inframe deletion	ACMG/ClinVar
9077delA	Gln3026Argfs*2	Q3026Rfs*2	23	2.78% (1)	OC	inframe deletion	ACMG

### BRCA1/2 germline VUS

*BRCA1/2* variants classified as VUS were found in 7.23% (n=38/525) of the total study population. Regarding *BRCA1*, eight different VUS were found, with an overall frequency in patients of 1.71% (n = 9/525). Most VUS were reported only in patients with OC (n= 7/8, 87.5%) with a low frequency per variant (n=1/9, 1.1%) and most of them occurred in exon 10. Two VUS had conflicting classifications between ClinVar and the ACMG guidelines: *BRCA1* c.93C>G (n = 2/9, 2.22%) and *BRCA1* c.287A>G (**Table S1**). The overall frequency of VUS in *BRCA2* was 5.52% (n=29/525). We identified 21 different VUS in *BRCA2* and the most frequent were c.5465A>T (n = 4, 13.79%), followed by c.3101T>C (n = 3, 10.34%), c.205C>A and c.437T>C (n = 2, 6.89%). Similarly, many of the VUS occurred in exon 11 (n = 9/21, 42.86%) and were reported only in patients with OC (n = 14/21, 66.67%). Variants c.2274T>G, c.3509C>T, c.7617 + 6T>C, c.7759C>T, and c.8462T>C had conflicting classifications between the ACMG guidelines and ClinVar (**Table S1**). In addition the pathogenic scores of VUS founds in this study are also presented in **Table S1**.

## Discussion

Breast and ovarian cancers are malignancies with a high incidence and prevalence in Latin American women. Costs of treatment and disability adjusted life years directly impact public health systems and society. The management of BC and OC is rapidly evolving with the discovery of new treatments that offer an improved survival advantage, as seen in recent years with PARP inhibitors in *BRCA1/2* mutation carriers (19)

There is limited information on the molecular epidemiology of BRCA1/2 in Peru. Although a third of the Peruvian population lives in Lima, there are economic and geographic barriers that make it difficult for patients to access adequate cancer care, including molecular tests and genetic counseling (20). Our work has some limitations, for example, the underrepresentation of patients from several regions of Peru, which have socioeconomic and ancestry differences with respect to Lima. Another limitation that could cause bias is that the study population was selected for possible treatment with PARP inhibitors (e.g., advanced-stage cancer, HER2-negative breast cancer).

The study of *BRCA1/2* variants in these patients is crucial to identify candidates for these treatments, but is also important to manage their future cancer risks. Patients with *BRCA1/2* mutations and their families can benefit from cancer risk reduction or early cancer detection strategies, such as double mastectomy or high-risk follow-up for BC and bilateral salpingo-oophorectomy for OC (12).

Latin American countries usually have less infrastructure and resources for genetic testing and treatment of patients with *BRCA1/2* than developed countries, and many of the cancer drugs approved in other countries are not available (21). Therefore, genetic testing is not always performed when needed. The interpretation of genetic variants can be more difficult in less-studied populations, and there are no specific open databases for variants in cancer-predisposing genes in Hispanic patients.

*BRCA1* c.2105dupT was the most frequently found variant in this study. It is located in the coding exon 10 and causes a translational frameshift with a predicted alternate stop codon. This alteration is expected to result in loss of function, according to ClinVar. Until now, this variant was previously reported in 6 of 44 (13.6%) mutated patients with ovarian cancer belonging to the Peruvian population. Furthermore, it was reported only once in the Canadian population. A similar variant, *BRCA1* c.2105dup, was identified in Caucasian and Hispanic individuals in a study that included only female carriers with pathogenic variants in *BRCA1*/2 *(* 22)

In our cohort, we observed the *BRCA1* c.68_69delAG mutation (185delAG), which occurs in exon 2 and creates a stop codon at position 39. This alteration leads to premature translation termination and significant protein truncation (23). Regarding its origin, it constitutes one of the primary founder mutations in the Ashkenazi Jewish population that arose 61 generations ago and was introduced into the Hispanic population 650 years ago (24). Currently, it has been reported among several other non-Jewish ethnic groups, such as Peruvians, Mexicans, Colombians, and Brazilians (25–28). A study in Peruvian patients diagnosed with breast cancer showed that 185delAG was the most common mutation found, observed in 7 of 13 carriers (54%). They also noted that two mutation carriers identified as of indigenous ancestry had the *BRCA1* 185delAG mutation (28). Unlike that study, we found that the mutation was observed in people born in Lima and Lambayeque. While it has not been determined whether they share an indigenous or Jewish ancestry, it is known that although the 185delAG mutation resides in a common haplotype among Ashkenazi Jews, it also arose independently in at least two non-Jewish populations, such as those in Malaysia and the United Kingdom. On the other hand, the *BRCA1* c.140G>T mutation is a missense variant, which occurs within exon 5 and the RING domain. It was previously reported as a VUS in Peruvian families with hereditary breast and ovarian cancer (18, 24). However, it was also identified in the Norwegian population as a pathogenic and non-founder mutation (29).

The *BRCA1* c.815_824dupAGCCATGTGG variant is located in exon 10 and is the most common pathogenic variant reported in African Americans (30). This pathogenic variant results in a frameshift and a spurious stop codon of 14 amino acids downstream. Due to its African origin, the variant was found in inherited patients with BC from Senegal and its allele frequency was 27.7%. In that study, the variant was also detected in a control population of sporadic BC cases and healthy controls without cancer (allele frequency estimated at 5% and 0.55%, respectively) (31). This variant was also found in Latin American populations, such as Colombian, Peruvian and Mexican (18, 32, 33). In **Table 4**, we summarize and compare our findings with those of other populations. Because we evaluated a very specific group of patients, comparison of prevalence of P/LP variants with other populations it would not be appropriate.

**Table 4:** Previous reports of *BRCA1/2* variants found in this study in different populations.

Most frequent BRCA 1/2 P/LP variants	Populations						
	Peruvian	Latin American	Asian	American	European	Jewish (Ashkenazi)	Reference
BRCA1 c.2105dupT	x	x		x			(22, 34)
BRCA1 c.68_69delAG	x	x	x	x	x	x	(22–25, 27, 33, 35–45)
BRCA1 c.140G>T	x	x	x	x			(18, 29, 36, 46–48)
BRCA1 c.815_824dupAGCCATGTGG	x	x	x	x	x		(18, 31, 33, 35, 49)
BRCA1 c.5123C>A	x	x			x		(28, 50–52)
BRCA1 c.1961delA	x		x				(28, 52)
BRCA1 c.2344dupA	None	None	None	None	None	None	
BRCA1 c.5251C>T					x		(53, 54)
BRCA 2 c.8023A>G		x	x		x		(48, 53–55)
BRCA2 c.6024dupG		x	x		x	x	(45, 55–60)
BRCA2 c.9235delG		x					(25)
BRCA2 c.1797_1801delTTATA		x					(61)
BRCA2 c.3395_3396delAA		x					(58)
BRCA2 c.9253dupA		x					(53)

In conclusion, in this selected Peruvian population, we found a frequency of 14.7% *BRCA1/2* germline mutations in BC and 20.7% in OC. The most recurrent mutations were *BRCA1* c.2105dupT and BRCA2 c.8023A>G. On the other hand, BRCA2 c.8023A>G, c.6024dupG and c.9235delG were not previously reported in Peruvian patients. BRCA1 c.2344dupA is a novel mutation that has not been previously reported in any database.

## Data availability statement

The datasets presented in this study can be found in online repositories. The names of the repository/repositories and accession number(s) can be found in the article/**Supplementary Material**.

## Ethics statement

The study was approved by the Institutional Review Board of the Universidad Privada San Juan Bautista (418-2021-CIEI-UPSJB) and conducted in compliance with all relevant ethical guidelines. Due to the retrospective nature of the study, the informed consent was waived.

## Author contributions

CB-L, JC, and FD conducted molecular assays and collected the data. CB-L, FD, YF, GR, and AC-R conducted analysis of variants. YF, GR, JA, LB, and JP conducted statistical analysis. MC conducted the bioinformatic analysis. YF, GR, JA, LB, and JP prepared **Tables 1**, **2**. YF, GR, JA, LB, and JP prepared **Figures 1**-**3**. All authors wrote the manuscript. All authors contributed to the article and approved the submitted version.
